# Defatted Rice Bran Supplementation in Diets of Finishing Pigs: Effects on Physiological, Intestinal Barrier, and Oxidative Stress Parameters

**DOI:** 10.3390/ani10030449

**Published:** 2020-03-08

**Authors:** Lijuan Fan, Ruihua Huang, Chengwu Wu, Yang Cao, Taoran Du, Guang Pu, Huan Wang, Wuduo Zhou, Pinghua Li, Sung Woo Kim

**Affiliations:** 1 Institute of Swine Science, Nanjing Agricultural University, Nanjing 210095, China; 18305177622@163.com (L.F.); rhhuang@njau.edu.cn (R.H.); wuchengwu05@163.com (C.W.); hudiebuhuifei@126.com (Y.C.); dtrnjau@163.com (T.D.); pigfarmer_pu@163.com (G.P.); 2017105010@njau.edu.cn (H.W.); zhouwuduo@njau.edu.cn (W.Z.); 2 Huaian Academy, Nanjing Agricultural University, Huaian 223003, China; 3 Industrial Technology System Integration Innovation Center of Jiangsu Modern Agriculture (PIG), Nanjing 210095, China; 4 Nanjing Agricultural University’s New Rural Research and Development Corporation of Huaian City, Huaian 223003, China; 5 Department of Animal Science, North Carolina State University, Raleigh, NC 27695, USA; sungwoo_kim@ncsu.edu

**Keywords:** defatted rice bran, blood, intestinal mucosal, oxidative stress, finishing pigs

## Abstract

**Simple Summary:**

Most studies on dietary fiber mainly focus on the digestibility of feed nutrients and microbial flora, etc. However, insufficient attention has been paid to the regulation of immune and oxidative stress of the intestinal tract by dietary fiber. This study investigated the effects of varying levels of defatted rice bran replacing corn on physiological, intestinal barrier, and oxidative stress parameters in finishing pigs. Based on the current findings, a high diet of rice bran will not only reduce the level of inflammatory factors in the peripheral blood of finishing pigs, but also enhance the healthy level of the colon through mucin2 and keap1-Nrf2 pathways. Our results can be used as reference for dietary rice bran to improve intestinal health in finishing pigs.

**Abstract:**

Rice bran is a waste product with low cost and high fiber content, giving it an added advantage over corn and soybean meal, which have to be purchased and always at a relatively higher cost. Under the background of increased attention to sustainable agriculture, it is significant to find alternative uses for this byproduct. A total of 35 finishing pigs were allotted to five dietary treatments: a control group with basal diet and four experimental diets where corn was equivalently substituted by 7%, 14%, 21%, and 28% defatted rice bran (DFRB), respectively. With increasing levels of DFRB, the neutrophil to lymphocyte ratio (NLR) linearly decreased (*p* < 0.05). In the jejunum, the mRNA level of nuclear factor erythroid-2 related factor-2 (*Nrf2*) exhibited a quadratic response (*p* < 0.01) with incremental levels of DFRB. In the colon, the mRNA levels of mucin 2 (*MUC2*), Nrf2, and NAD(P)H: quinone oxidoreductase 1 (*NQO1*) were upregulated (linear, *p* < 0.05) and heme oxygenase-1 (*HO-1*) was upregulated (linear, *p* < 0.01). Overall, using DFRB to replace corn decreased the inflammatory biomarkers of serum and showed potential function in modulating the intestinal barrier by upregulating the mRNA expression levels of *MUC2* and downregulating that of *Nrf2*, *NQO1*, and *HO-1* in the colon.

## 1. Introduction

Corn and soybean meal are the main feedstuffs for pigs, providing balanced nutrients [1]. With increasing demands for conventional feedstuffs by pig production, more attention is paid to alternative and locally available feedstuffs.

Rice bran is one of the co-products of rice processing and is a combined layer of pericarp, seed coat, aleurone layer, and embryo [2]. The decreasing cost of rice production and increasing supply of corn make rice bran an appealing candidate for alternative feedstuffs. However, rice bran contains lipoxygenases that break down the fatty acids present in the bran, which would eventually result in a detrimental effect on its flavor [3]. In order to prevent rice bran from oxidation, defatted rice bran (DFRB) has been used in practical production.

Rice bran includes several bioactive components such as pectin, arabinoxylan, lignin, cellulose, hemicellulose, β-glucan, and gum [4,5] have been suggested to be functional polysaccharides with immunomodulatory properties [6]. Meanwhile, the polysaccharide is reported as a kind of effective free radical scavenger and antioxidant, playing a critical role in protecting against oxidation damage in living organisms [7]. The health prospects of rice bran have attracted increasing interest. Potential health benefits of dietary fiber associated with mucosal immune modulation have been shown in recent researches [8,9].

A previous study by our team proved that dietary supplementation of DFRB had no effect on the growth performance of Suhuai finishing pigs [10]. As the most important immune organs, the intestinal tract is the first line of defense to protect the homeostasis of the body’s internal environment [11] and the function of the intestinal mucosal barrier is a direct manifestation of intestinal health [12]. At present, insufficient attention has been paid to the regulation of immune and oxidative stress of the intestinal tract by DFRB. We hypothesized that moderate levels of DFRB might improve intestinal barrier function and modulate the steady state of redox reaction.

This study aimed to evaluate the effects of dietary supplementation of DFRB on physiological, intestinal barrier, and oxidative stress parameters in finishing pigs, providing reference for the reasonable addition of DFRB in the diet of finishing pigs.

## 2. Materials and Methods

The protocol of this experiment was reviewed and approved by the Nanjing Agricultural University Animal Welfare and Ethics Committee (Certification No.: SYXK (Su) 2017-0007), following the requirements of the Regulations for the Administration of Affairs Concerning Experimental Animals.

### 2.1. Experimental Animals and Design

The experimental animals were from Huaiyin pig breeding plant in Jiangsu Province, China. Suhuai pig is a synthetic Chinese breed that is derived from Large white pig (75%) and Chinese Huai pig (25%) [13]. A total of 35 Suhuai finishing pigs (158.5 ± 2.0 d) with body weight (BW) of 62.9 ± 0.8 kg were selected and allotted to 5 treatment groups (7 replicates for each treatment) using a randomized complete block design. The 5 dietary treatments included a basal diet without supplementation of DFRB and 4 experimental diets with supplementation of 7%, 14%, 21%, and 28% DFRB, respectively.

### 2.2. Diets and Feeding Management

The basal diet was configured according to the Feeding Standard of Swine (NY/T 65-2004) 60–90 kg Standard of Meat-fat Type Growing-finishing Pig. The basal diet and the four experimental diets were formulated to be nearly equal in nutritional components except for dietary fiber (DF) content (Table 1). Pigs from each treatment were individually housed in pens which were equipped with the Osborne Testing Stations System (OTSS) and water saving type stainless-steel drinker to allow the pigs access to feed and water ad libitum. All the groups were fed a basal diet during the 10-d preliminary trial period. Subsequently, different treatment groups were fed with corresponding diets for 28 days. All pigs were housed in a temperature-controlled room with partially slatted floors. Over the whole trial period, the experimental animals were in good health.

### 2.3. Sample Collection

Two blood samples were collected from the jugular vein of each pig at slaughtering. One was collected into glass tubes without anticoagulant. After centrifugation (1000× *g* for 15 min at 4 °C), serum samples were collected and stored at −80 °C for determination of serum biochemistry and serum immunoglobulins. The other was collected into a vacutainer containing ethylene diamine tetraacetic acid (EDTA). The vacuum tubes were immediately placed in an icy box until they were sent to the veterinary hospital (within 2 h) for the examination of blood cell counts. Segments (5 cm in length) of jejunum (the middle part of the distance from pylorus to ileocecal valve) and colon (the middle part of colon) were intercepted. All the segments were opened longitudinally and washed with saline solution to remove contents. The intestinal mucosa samples were collected and rapidly frozen in liquid nitrogen until analysis.

### 2.4. Blood Sample Analysis

Routine blood test was submitted to Mairui 5300 Blood Cell Analyzer (Wuhan Shengshida Medical Equipment Co., Ltd., Wuhan, China). Serum samples were analyzed for total protein (TP), albumin (ALB), globulin (GLOB), alkaline phosphatase (ALP), glutamate pyruvic transaminase (GPT), glucose (GLC), blood urea nitrogen (BUN), amylase (vAMY), total cholesterol (THOL), and triglyceride (TC) using a Beckman 480 Biochemical Analyzer (Beckman Coulter Commercial Enterprise (China) Co., Ltd., Shanghai, China). Porcine-specific ELISA kits were used to quantify immunoglobulin A (IgA), immunoglobulin G (IgG), and immunoglobulin M (IgM) in accordance with the manufacturer’s instructions (Wuhan Huamei Bioengineering Co., Ltd., Wuhan, China). All samples were analyzed in duplicate.

### 2.5. Assays of Secretory Immunoglobulin A (SIgA), IgM, and Cytokines Concentration

After homogenization of mucosa samples in tissue homogenate diluent and centrifugation at 5000× *g* for 5 min at 4 °C, the supernatants were used for determination of SIgA (ug/mg), IgM (ug/mg), and inflammatory cytokines (pg/mg) using commercially available ELISA kits (Wuhan Huamei Bioengineering Co., Ltd., Wuhan, China; Abcam, Cambridge, MA, USA; and R&D Systems, Inc., Minneapolis, MN, USA, respectively) according to the manufacturer’s procedures. The total protein content of supernatant liquid was determined using the bicinchoninic acid (BCA) method. Concentrations of SIgA, IgM, and cytokine were standardized to the protein in each sample.

### 2.6. RNA Extraction and Gene Expression Analysis

Total RNA from the mucosa of jejunum and colon were extracted using TRIzol reagent (Shanghai Yuanye Biotechnology Co., Ltd., Shanghai, China) according to the manufacturer’s instructions after the samples were ground with liquid nitrogen. The integrity of RNA was checked by 1% formaldehyde agarose gel electrophoresis. The concentration and purity of mRNA were measured spectrophotometrically (Shanghai Meixi Instrument Co., Ltd., Shanghai, China). Those RNA samples with an OD260:OD280 ratio (where OD is the optical density) ranging from 1.8 to 2.0 were eligible for use in subsequent experiments. Reverse transcription reactions were performed using a RT reagent kit (Applied Biological Materials (ABM) Inc) following the manufacturer’s instructions. The generated cDNA was immediately used for real-time fluorescence quantitative PCR analysis. The mRNA expression levels of mucin 2 (*MUC2*), porcine beta defensin 1 (*PBD1*), proline-arginine-rich antimicrobial peptides (PR39), nuclear factor erythroid-2 related factor-2 (*Nrf2*), NAD(P)H: quinone oxidoreductase 1 (*NQO1*), and heme oxygenase-1 (*HO-1*) in the mucosa of jejunum and colon were analyzed by real-time quantitative PCR with SYBR Green PCR reagents (ABM). The sequences of primers (Table 2) used in the experiment were synthesized by Tsingke Biotech Co., Ltd. (Beijing, China). The reaction was performed using the following cycle program: A hold stage at 95 °C for 10 min; 40 cycles for PCR stage at 95 °C for 15 s and at 60 °C for 60 s; a melt curve stage at 95 °C for 15 s, at 60 °C for 60 s, and at 95 °C for 1 s.

A melting curve analysis was generated following each real-time quantitative PCR so as to check and verify the specificity and purity of all PCR products. The reference gene glyceraldehyde-3-phosphate dehydrogenase (GAPDH) was used for normalization, and the relative mRNA expression levels of the target gene in comparison with the reference gene were calculated using the 2^−ΔΔCT^ method. Each sample was run simultaneously in triplicate on the same PCR plate.

### 2.7. Statistical Analysis

This study used a randomized complete design. DFRB was the main effect. Each pig was considered as the experimental unit for all analyses. All data were submitted to one-way ANOVA procedure of SPSS 25.0 software. The significance of linear and quadratic responses was declared at *p* < 0.05 and a statistical trend was considered for 0.05 ≤ *p* < 0.10. The results were presented as the means and standard error of the means (SEM).

## 3. Results

### 3.1. Blood Cell Counts

Table 3 reported the effects of varying DFRB levels on blood cell counts. With the DFRB level increased in the diets, neutrophils percentage decreased (quadratic, *p* < 0.01), lymphocytes percentage linearly increased (*p* < 0.05), and neutrophil to lymphocyte ratio (NLR) linearly decreased (*p* < 0.05). The other indexes were not affected by the supplementation levels of DFRB.

### 3.2. Serum Biochemistry Parameters

The results of serum biochemistry parameters are reported in Table 4 and Table 5. With the DFRB level increased in the diets, the level of serum albumin and amylase tended to increase linearly (*p* = 0.087 and *p* = 0.094, respectively). There was no difference in other serum biochemistry parameters. Serum immunoglobulins (Ig) did not change with increasing levels of DFRB.

### 3.3. Intestinal Immune Barrier

Table 6 and Table 7 report the effects of varying DFRB levels on intestinal immune barrier-related indexes. The levels of interferon γ (IFN-γ), interleukin 1β (IL-1β), interleukin 6 (IL-6), interleukin 10 (IL-10), interleukin 12 (IL-12), SIgA, and IgM neither exhibited a linear nor quadratic response to the diets (*p* > 0.05).

### 3.4. Intestinal Chemical Barrier

The mRNA expression levels of intestinal chemical barrier-related genes in the mucosa of jejunum and colon of finishing pigs are presented in Table 8. In the jejunum, the supplementation levels of DFRB had no effect on the mRNA expression levels of *MUC2*, *PBD1*, and *PR39*. In the colon, the mRNA levels of *MUC2* was upregulated (linear, *p* < 0.05; quadratic, *p* < 0.05) with increasing levels of DFRB in the diet. There was a tendency (linear, *p* = 0.057) that the mRNA expression level of *PR39* was increased as the concentration of DFRB increased in the diets. However, the mRNA expression level of *PBD1* showed no response to these diets.

### 3.5. Oxidative Stress Index

Table 9 shows the effects of increasing DFRB levels on the mRNA expression levels of oxidative stress indexes in the mucosa of jejunum and colon. In the jejunum, the mRNA level of *Nrf2* exhibited a quadratic response (*p* < 0.05) with incremental levels of DFRB. There was a tendency (*p* = 0.059 and *p* = 0.081, respectively) for the mRNA expression levels of *NQO1* and *HO-1* to show a quadratic response as the concentration of DFRB increased in the diets. In the colon, the mRNA levels of *Nrf2* and *NQO1* were downregulated (linear, *p* < 0.05) and *HO-1* was downregulated (linear, *p* < 0.01; quadratic, *p* < 0.05) with increasing levels of DFRB in the diets. *NQO1* tended (*p* = 0.054) to show a quadratic response with the DFRB levels in the diet.

## 4. Discussion

In the current trial, the diets were formulated in terms of the Feeding Standard of Swine (NY/T 65-2004) 60–90 kg Standard of Meat-fat Type Growing-finishing Pig, since Suhuai pig is a synthetic breed. The five experimental diets were supplemented with 0%, 7%, 14%, 21%, and 28% DFRB, respectively. In order to balance digestible energy and amino acids among five groups, we changed corn, wheat bran, soybean oil, lysine, etc. in four experimental diets. In this case, it led to small differences in the composition of other components in addition to dietary fiber components. In view of the fact that the level of DFRB is the main difference of dietary components among groups, the differences in the indicators were mainly caused by the different levels of dietary fiber of DFRB.

Blood as an easily accessible organ system is often used to screen for pathological conditions. Neutrophils are formed within the bone marrow during hematopoiesis [18]. Researchers once believed that neutrophils were present only during the acute phase of the inflammatory response, functioning only as pathogen killers. Now it is known that neutrophils can shape the immune landscape by communicating with macrophages, dendritic cells, and cells of the adaptive immune response through direct cell–cell contact or as soluble mediators [19,20,21]. Neutrophil homeostasis is likely influenced by phagocytic uptake in the periphery, cell mass in the bone marrow, and inflammatory or pathogen challenge [22]. Another study concluded that rice bran induced an anti-inflammatory environment that helped to protect against tumorigenesis [23]. The linear and quadratic response in neutrophils percentage resulting from varying supplementation levels of DFRB indicates that DFRB may reduce the number of neutrophils by inhibiting the inflammatory environment.

Lymphocytes can be divided into T lymphocyte, B lymphocyte, and natural killer (NK) cells according to its migration, surface molecule, and function, which mediate cellular immunity, humoral immunity, killing tumor cells, and virus-infected cells, respectively. In our experiments, the linear response to lymphocytes percentage resulting from varying supplementation levels of DFRB indicated that DFRB improved immune status.

NLR has been postulated as a marker of systemic inflammation [24,25]. It has been shown to be associated with a variety of malignancies [26]. Swine serves as an important biomedical model for various human diseases because the immune system of pigs is very similar to that of human beings. In human medicine, the sum of lymphocyte percentage and neutrophil percentage is almost 100%; the decrease of neutrophil percentage will inevitably lead to the increase of lymphocyte percentage and the decrease of NLR. In our experiments, our experimental data followed this biological law.

In this study, there was a trend that amylase and albumin increased linearly with increasing levels of DFRB supplementation in the diets. The change of supplementation levels of DFRB had no effect on the content of globulin and total protein. The results of globulin in blood were consistent with the results of serum immunoglobulins. We inferred that the DFRB did not change the content of serum protein. This observation was consistent with results of previous experiment [27].

Cytokines play a crucial role in immune response, and their balance is important for preventing infection [28]. In the present experiment, no difference was observed in the concentrations of cytokines in response to the supplementation level of DFRB. The reason for this observation may be that there were not enough immunological stimuli to induce changes in the concentrations of pro-inflammatory cytokines since pigs used in this experiment were of good health status.

Mucins, secreted by goblet cells in the epithelium, are the determining constituents of the mucus layer. In the small and large intestine, the secreted mucus is predominantly composed of *MUC2* [29], which forms a considerable chemical barrier to enteric commensals and pathogens so that absence of the secreted *MUC2* leads to colitis [30]. Our results showed that there was an increased expression of *MUC2* in the colon in response to the increased DFRB supplementation. According to the research of the former scholars that dietary treatment was reported to affect mucus secretion [31], we speculate that DFRB increased the expression of *MUC2* and had a positive effect on intestinal health. The reason for the quadratic may be due to the relatively small value of the 21% group. This suggests that there is not always a positive correlation between the level of DFRB supplementation and intestinal health. It may have adverse effects on intestinal health when the proportion of DFRB exceeds the appropriate range.

Nrf2 has been proven to be a crucial transcription factor that protects organism from oxidants [32]. Nrf2 locates in the cytoplasm as it is negatively controlled by cytoplasmic Kelch-like ECH-associated protein 1 (Keap-1) under homeostatic conditions. Reactive oxygen species (ROS) play a key role in the activation of *Nrf2* [33,34] under the stimulation of ROS, Nrf2 translocates into the nucleus, where it combines with antioxidant response elements (AREs) and promotes the transcription of antioxidant proteins, including *NQO1* and *HO-1* [35]. For a long time, the Keap1-*Nrf2* pathway has been researched for clinical applications and to improve human health [36]. The antioxidant effect of dietary fiber is based on the polyphenol compounds bound to polysaccharide complexes, which are released in the gut and function as antioxidants [37]. In this experiment, dietary fiber in defatted rice bran may play a direct role in antioxidation, which may be account for the decreased expression levels of *Nrf2*, *NQO1*, and *HO-1* in the colon.

Distinct microbial communities between the small intestine and large intestine were found [38,39]. The small intestine is mainly responsible for food digestion and absorption, while the large intestine, which has lots of microorganisms, is related to microbial fermentation [40]. In the present experiment, the mRNA expression level of *Nrf2* in jejunum exhibited a quadratic response with incremental levels of DFRB. In the colon, the mRNA levels of *Nrf2* were downregulated linearly with increasing levels of DFRB in the diets, which may be due to the different fermentation ability of different intestinal segments to fiber. Our results showed that it is meaningful to understand the differential metabolic capacities among distinct intestinal locations. It will contribute to informing strategies to improve gut health based on defatted rice bran addition in the diets of finishing pigs.

## 5. Conclusions

This study showed that DFRB can be used as a feedstuff for finishing pigs. Dietary supplementation of DFRB decreased the inflammatory biomarkers of serum and showed the potential function to modulate the intestinal barrier by upregulating the mRNA expression levels of *MUC2* and downregulating that of *Nrf2*, *NQO1*, and *HO-1* in the colon.

## Figures and Tables

**Table 1:** Composition of experimental diets.

Items	Defatted Rice Bran (DFRB), %				
	0	7	14	21	28
Ingredients (%)					
Corn	68.61	62.00	55.00	48.00	41.00
Wheat bran	15.40	15.80	16.15	16.67	17.21
DFRB	0.00	7.00	14.00	21.00	28.00
Soybean meal	13.30	11.70	10.40	8.95	7.50
Soybean oil	0.00	0.84	1.83	2.78	3.74
98.5% Lysine	0.03	0.04	0.03	0.03	0.03
Salt (NaCl)	0.30	0.30	0.30	0.30	0.30
Limestone	0.82	0.85	0.85	0.85	0.85
CaHPO4	0.75	0.68	0.65	0.63	0.58
60% Choline chloride	0.04	0.04	0.04	0.04	0.04
Premix ^1^	0.40	0.40	0.40	0.40	0.40
Measured composition ^2^					
Dry matter (DM, %)	88.56	88.68	88.93	89.16	88.46
Crude protein (CP, %)	15.60	16.67	16.13	15.73	16.40
Crude fiber (CF, %)	4.38	4.72	5.06	5.38	5.58
Insoluble detergent fiber (IDF, %)	16.14	17.19	18.42	19.32	23.37
Soluble detergent fiber (SDF, %)	0.52	0.56	0.68	0.73	0.82
Acid detergent fiber (ADF, %)	5.53	6.25	6.53	7.08	8.13
Neutral detergent fiber (NDF, %)	8.89	11.80	12.93	14.35	17.94
Ether extract (EE, %)	5.19	5.08	5.32	5.27	5.38
Hemicellulose (%)	3.80	5.69	7.09	8.00	10.34
Cellulose (%)	4.06	4.43	4.71	5.09	5.79
Acid detergent lignin (ADL, %)	0.46	0.54	0.72	0.96	1.13
Calculated composition ^2^					
Metabolic energy (MJ, %)	12.13	12.13	12.22	12.27	12.31
Calcium (%)	0.55	0.55	0.55	0.55	0.55
Available phosphorus (%)	0.27	0.27	0.27	0.27	0.27
L-lysine (%)	0.65	0.65	0.65	0.66	0.65
Methionine + cystine (%)	0.45	0.45	0.46	0.47	0.47
Total detergent fiber (TDF, %)	16.70	17.75	19.10	20.05	24.11

^1^ The premix provided the following per kg of diets: vitamin A 8000 international unit (IU), vitamin D3 1500 IU, vitamin E 100 mg, vitamin K3 4 mg, vitamin B1 2 mg, vitamin B2 8 mg, vitamin B6 3 mg, vitamin B12 0.04 mg, niacin 30 mg, pantothenic acid 35 mg, folic acid 0.6 mg, biotin 0.13 mg, choline 150 mg, Fe 60 mg, Cu 5 mg, Zn 60 mg, Mn 10 mg, Se 0.15 mg, I 0.1 mg. ^2^ DM, CP, CF, IDF, SDF, ADF, NDF, EE, hemicellulose, cellulose, and ADL were measured values, whereas the other nutrient levels were calculated values.

**Table 2:** List of primers used in this study.

Gene	Primer Sequences (5 ′-3 ′) ^1^	Reference
*GAPDH*	F: GAAGGTCGGAGTGAACGGAT R: CATGGGTAGAATCATACTGGAACA	[14]
*MUC2*	F: CTGCTCCGGGTCCTGTGGGA R: CCCGCTGGCTGGTGCGATAC	[15]
*PBD1*	F: GGCCCTTGAGGATGTGATAAA R: CTGTGGGCATGTCACTTAGAT	[16]
*PR39*	F: CTTCCCAGTAGAGGCATGTTATT R: GCCACAGTTTGAGGTGATTTG	[16]
*Nrf2*	F: CGTGAAGCGACTGAACCT R: ATGTAGCCGAAGAACCT	[17]
*NQO1*	F: TGCCTTCCTTGACTTGCT R: TCCCGGCTTTACATCCTA	[17]
*HO-1*	F: TTCACCTTCCCGAGCAT R: GCCTCTTCTGTCACCCTGT	[17]

^1^ F = forward primer; R = reverse primer.

**Table 3:** Effects of increasing defatted rice bran (DFRB) supplementation on blood cell counts of finishing pigs ^1^.

Item	DFRB, %					SEM	*p*-Value	
	0	7	14	21	28		Linear	Quadratic
Leukocyte count (10^9^ L^−1^)	24.07	24.69	23.51	21.90	24.04	0.75	0.594	0.774
Neutrophil granulocyte count (10^9^ L^−1^)	12.60	12.87	12.12	10.90	11.56	0.49	0.101	0.350
Neutrophils percentage%	52.35	52.13	51.55	49.77	48.09	1.10	0.014	0.007
Lymphocytes count (10^9^ L^−1^)	10.27	10.72	10.40	9.73	11.23	0.43	0.667	0.752
Lymphocytes percentage%	42.67	43.42	44.24	44.43	46.71	1.19	0.016	0.062
Mononuclear cells count (10^9^ L^−1^)	0.67	0.64	0.56	0.79	0.79	0.05	0.612	0.665
NLR ^2^	1.22	1.22	1.21	1.11	1.07	0.05	0.030	0.062
Mononuclear cells percentage%	2.78	2.59	2.38	3.61	3.29	0.17	0.250	0.475
Eosinophils count (10^9^ L^−1^)	0.27	0.24	0.24	0.26	0.26	0.03	0.994	0.365
Eosinophils percentage%	1.12	0.97	1.02	1.19	1.08	0.10	0.908	0.661
Basophilic granulocyte count (10^9^ L^−1^)	0.26	0.21	0.19	0.21	0.20	0.02	0.186	0.155
Basophilic granulocyte percentage%	1.08	0.85	0.81	0.96	0.83	0.06	0.311	0.582
Platelet count (10^9^ L^−1^)	248.68	202.88	239.78	234.40	199.00	15.01	0.419	0.759
Mean platelet volume (fL)	8.07	8.00	7.44	7.89	8.06	0.13	0.900	0.419
Platelet distribution width	15.18	15.27	15.17	15.07	15.18	0.06	0.452	0.796
Thrombocytocrit	0.20	0.16	0.18	0.19	0.16	0.01	0.456	0.799
Red blood cell count (10^12^ L^−1^)	7.53	7.69	7.80	7.28	7.76	0.14	0.953	0.999
Hematocrit	44.53	45.29	45.31	42.95	45.29	0.88	0.838	0.973
Mean corpuscular volume (fL)	59.28	58.86	58.22	58.91	58.44	0.63	0.266	0.462
Hemoglobin concentration (g L^−1^)	130.55	137.45	137.36	127.12	138.02	2.22	0.814	0.975
Mean erythrocyte hemoglobin Concentration (g L^−1^)	293.33	304.12	302.94	298.05	304.69	2.00	0.344	0.595
Average hemoglobin content of red blood cell (pg)	17.41	17.88	17.63	17.51	17.80	0.14	0.586	0.865
Red blood cell distribution width coefficient of variation	17.05	17.24	16.91	16.68	18.14	0.36	0.440	0.398
Red blood cell distribution width-standard deviation	42.18	41.93	41.80	42.67	39.67	0.69	0.300	0.386

^1^ Values are means and pooled SEMs; SEM, standard error of mean; *n* = 7. ^2^ NLR, neutrophil to lymphocytes ratio.

**Table 4:** Effects of increasing DFRB supplementation on serum biochemistry parameters of finishing pigs ^1^.

Item	DFRB, %					SEM	*p*-Value	
	0	7	14	21	28		Linear	Quadratic
Total protein (g L^−1^)	76.33	76.46	74.16	76.18	73.16	0.93	0.192	0.485
Albumin (g L^−1^)	33.44	34.89	35.26	34.67	35.95	0.56	0.087	0.287
Globulin (g L^−1^)	42.82	41.29	39.49	42.24	36.66	1.08	0.168	0.442
A/G ^2^	0.83	0.86	0.88	0.82	1.00	0.32	0.229	0.403
Glutamic pyruvic transaminase (U L^−1^)	63.06	68.71	59.70	65.19	59.01	2.57	0.437	0.696
Alkaline phosphatase (U L^−1^)	110.79	132.81	129.00	125.68	132.91	6.81	0.243	0.407
Urea (mmol L^−1^)	5.90	7.03	6.95	5.78	6.40	0.24	0.913	0.757
Amylase (U L^−1^)	1456.86	2318.04	2050.74	2210.01	2611.04	136.07	0.094	0.315
Glucose (mmol L^−1^)	4.66	5.71	4.46	4.69	5.33	0.25	0.878	0.963
Total cholesterol (mmol L^−1^)	2.61	2.63	2.63	2.78	2.71	0.07	0.125	0.399
Triglyceride (mmol L^−1^)	0.55	0.64	0.62	0.41	0.72	0.04	0.811	0.895

^1^ Values are means and pooled SEMs; SEM, standard error of mean; *n* = 7. ^2^ A/G, albumin/globulin.

**Table 5:** Effects of increasing DFRB supplementation on serum immunoglobulins of finishing pigs ^1^.

Item ^2^	DFRB, %					SEM	*p*-Value	
	0	7	14	21	28		Linear	Quadratic
IgA (μg/mL)	187.78	208.27	165.68	354.75	232.41	30.37	0.391	0.741
IgM (μg/mL)	171.69	151.28	307.30	95.32	146.92	22.69	0.738	0.815
IgG (μg/mL)	2117.57	1953.98	1802.16	678.50	1552.05	218.16	0.214	0.481

^1^ Values are means and pooled SEMs; SEM, standard error of mean; *n* = 7. ^2^ IgA, immunoglobulin A; IgM, immunoglobulin M; IgG, immunoglobulin G.

**Table 6:** Effects of increasing DFRB supplementation on SIgA and IgM in jejunum and colon of finishing pigs ^1^.

Item ^2^	Intestinal Segment	DFRB, %					SEM	*p*-Value	
		0	7	14	21	28		Linear	Quadratic
SIgA (ug/mg)	Jejunum	2.01	1.14	1.20	1.04	1.15	0.23	0.165	0.130
	Colon	1.11	3.19	1.38	0.92	1.17	0.33	0.283	0.247
IgM (ug/mg)	Jejunum	6.00	3.50	2.50	3.96	4.69	0.53	0.673	0.108
	Colon	5.59	4.48	4.72	5.44	5.12	0.65	0.991	0.656

^1^ Values are means and pooled SEMs; SEM, standard error of mean; *n* = 7. ^2^ SIgA, secretory immunoglobulin A; IgM, immunoglobulin M.

**Table 7:** Effects of increasing DFRB supplementation on cytokines in jejunum and colon of finishing pigs ^1^.

Item ^2^	Intestinal Segment	DFRB, %					SEM	*p*-Value	
		0	7	14	21	28		Linear	Quadratic
IL-10 (pg/mg)	Jejunum	5.77	7.17	6.53	14.17	5.32	1.04	0.160	0.716
	Colon	1.73	1.13	1.73	1.32	1.49	0.09	0.778	0.896
IL-12 (pg/mg)	Jejunum	26.60	18.00	23.56	41.03	28.33	3.56	0.400	0.755
	Colon	29.32	16.15	24.01	16.67	16.37	2.35	0.210	0.486
IL-6 (pg/mg)	Jejunum	0.21	0.40	0.21	0.15	0.53	0.06	0.520	0.667
	Colon	1.70	0.92	0.65	1.99	1.15	0.16	0.989	0.870
IL-1β (pg/mg)	Jejunum	51.00	34.12	32.27	82.31	34.56	4.52	0.855	0.983
	Colon	60.62	42.43	56.03	56.09	46.56	3.49	0.620	0.904
IFN-γ (pg/mg)	Jejunum	142.62	103.00	78.45	227.01	82.95	13.64	0.985	0.994
	Colon	49.51	44.82	52.42	49.14	43.76	2.97	0.603	0.690

^1^ Values are means and pooled SEMs; SEM, standard error of mean; *n* = 7. ^2^ IL-10, interleukin 10; IL-12, interleukin 12; IL-6, interleukin 6; IL-1β, interleukin 1β; IFN-γ, interferon γ.

**Table 8:** Effects of increasing DFRB supplementation on *MUC2*, *PBD1*, and *PR39* in jejunum and colon of finishing pigs ^1^.

Item ^2^	Intestinal Segment	DFRB, %					SEM	*p*-Value	
		0	7	14	21	28		Linear	Quadratic
*MUC2*	Jejunum	1.00	0.93	1.23	0.76	1.31	0.11	0.383	0.408
	Colon	1.00	1.45	2.03	0.73	3.42	0.20	0.019	0.028
*PBD1*	Jejunum	1.00	3.07	3.71	3.82	1.85	0.74	0.501	0.341
	Colon	1.00	2.33	2.67	4.22	2.09	0.66	0.290	0.367
*PR39*	Jejunum	1.00	13.38	12.64	0.29	10.94	3.09	0.643	0.709
	Colon	1.00	1.26	6.57	0.77	12.78	1.79	0.057	0.104

^1^ Values are means and pooled SEMs; SEM, standard error of mean; *n* = 7. ^2^ MUC2, mucin 2; PBD1, porcine beta defensin 1; PR39, proline-arginine-rich antimicrobial peptides.

**Table 9:** Effects of increasing DFRB supplementation on *Nrf2*, *NQO1*, and *HO-1* in jejunum and colon of finishing pigs ^1^.

Item ^2^	Intestinal Segment	DFRB, %					SEM	*p*-Value	
		0	7	14	21	28		Linear	Quadratic
*Nrf2*	Jejunum	1.00	1.06	1.40	1.24	0.68	0.10	0.490	0.039
	Colon	1.00	1.22	0.96	0.62	0.68	0.07	0.010	0.447
*NQO1*	Jejunum	1.00	0.43	0.55	0.64	0.63	0.07	0.226	0.059
	Colon	1.00	0.81	0.52	0.44	0.66	0.07	0.025	0.054
*HO-1*	Jejunum	1.00	0.39	0.46	0.42	0.47	0.09	0.079	0.081
	Colon	1.00	0.47	0.34	0.38	0.30	0.08	0.003	0.047

^1^ Values are means and pooled SEMs; SEM, standard error of mean; *n* = 4. ^2^ *Nrf2*, nuclear factor erythroid-2 related factor-2; *NQO1*, NAD(P)H: quinone oxidoreductase 1; *HO-1*, heme oxygenase-1.
